# Integrative respiratory support during thoracoabdominal aortic aneurysm repair in a patient with severe lung disease: a case report

**DOI:** 10.1186/s44215-023-00040-7

**Published:** 2023-05-10

**Authors:** Ryota Hara, Joji Ito, Hidetaka Onodera, Minoru Tabata

**Affiliations:** 1 Department of Cardiovascular Surgery, Tokyo Bay Urayasu Ichikawa Medical Center, Urayasu, Japan; 2 Department of Anesthesiology, Tokyo Bay Urayasu Ichikawa Medical Center, Urayasu, Japan; 3 Department of Cardiovascular Surgery, Juntendo University Graduate School of Medicine, 2-1-1, Hongo, Bunkyo-Ku, Tokyo, 113-8421 Japan

**Keywords:** Thoracoabdominal aortic aneurysm repair, Severe chronic obstructive pulmonary disease, One-and-a-half lung ventilation, Partial bronchial blocking, Venovenous extracorporeal membrane oxygenation

## Abstract

**Background:**

Thoracoabdominal aneurysm repair is a highly complicated procedure, especially among patients with severe lung disease, resulting in respiratory problems during and after the surgery. Herein, we designed a novel intraoperative respiratory support to address this.

**Case presentation:**

An open thoracoabdominal aortic aneurysm repair was performed on a 65-year-old man who had severe chronic obstructive pulmonary disease with a giant right lung bulla. One-and-a-half lung ventilation by left lower lobe blockade was maintained during the operation to avoid right barotrauma. Cardiopulmonary bypass (CPB) was established with venous cannulas in the right internal jugular vein and left femoral vein for elective venovenous extracorporeal membrane oxygenation (VV-ECMO). After aortic repair and withdrawal from CPB, the VV-ECMO was consecutively initiated using the same circuit by connecting the arterial cannula to the right internal jugular venous cannula. The patient maintained adequate oxygenation during hemostasis under the support of VV-ECMO after protamine was administered. He was weaned from VV-ECMO in the operating room and discharged without any complications.

**Conclusion:**

Partial bronchial blockage and intraoperative VV-ECMO using the same circuit of CPB were useful methods for severe lung disease during a thoracoabdominal aortic repair.

## Background

In the field of cardiovascular surgery, thoracoabdominal aortic repair is one of the most invasive procedures and has high mortality and morbidity rates [1]. The surgery is more complicated among patients with severe lung disease because left lung deflation is required for optimal exposure of the aorta. One-lung ventilation could fail to maintain adequate oxygenation and cause respiratory complications in such patients [2]. To solve this problem, we designed an original intraoperative respiratory support system using a bronchial blocker and venovenous extracorporeal membrane oxygenation. Herein, we report a case of thoracoabdominal aortic repair with integrative respiratory support in a patient with a thoracoabdominal aortic aneurysm and severe lung disease.

## Case presentation

**Fig. 1:**
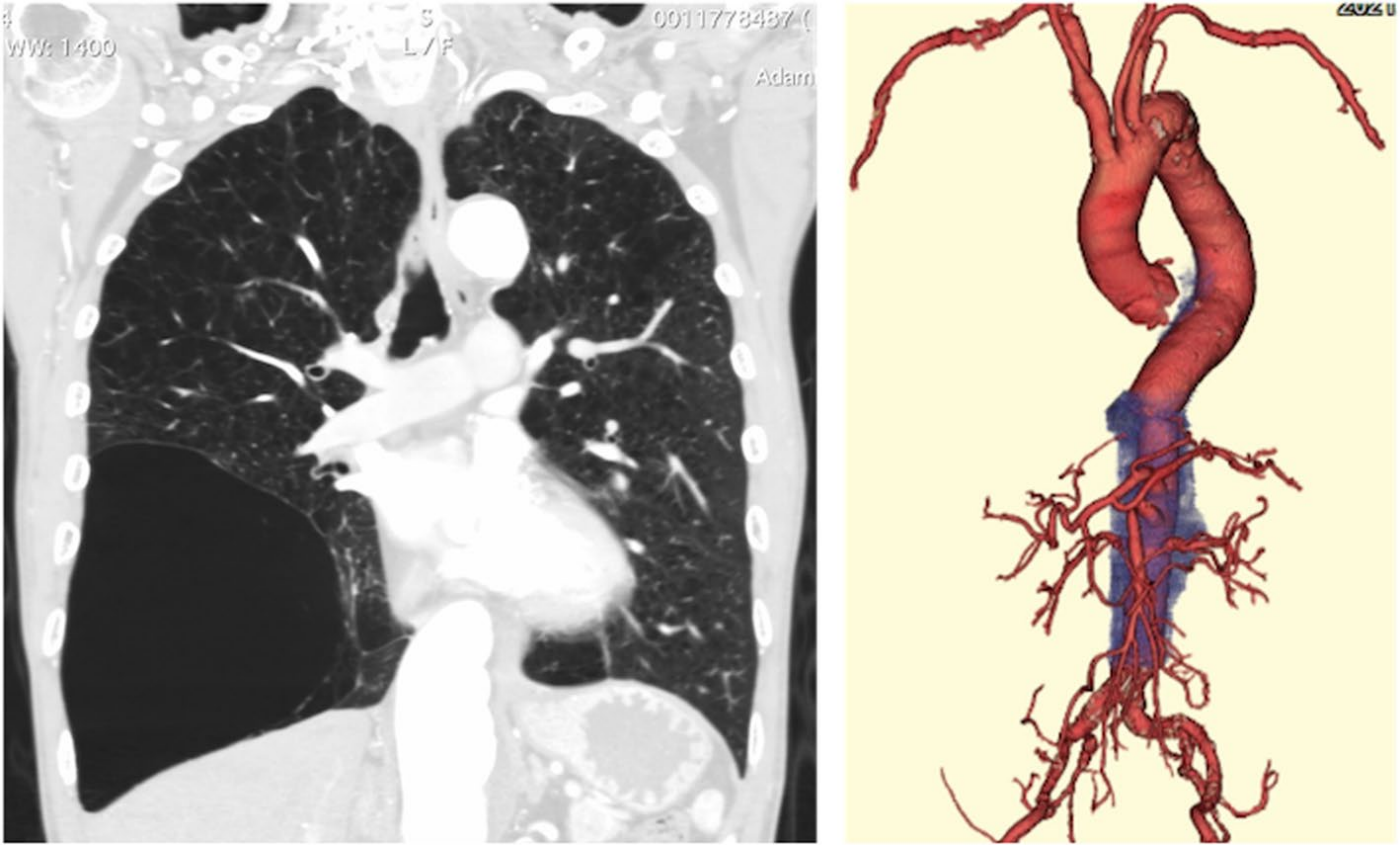
The patient’s preoperative computed tomographic findings. Left: a giant bulla in the right lung. Right: three-dimensional image of the thoracoabdominal aneurysm

A 65-year-old man with Crawford type III thoracoabdominal aortic aneurysm (atherosclerotic) was referred to our hospital [3]. On serial computed tomography (CT), the aneurysm was atherosclerotic (Fig. 1), the maximal diameter was 54 mm, and it progressively dilated by 12 mm in 3 years. Considering the patient’s body size (height, 169.0 cm, and weight, 56.0 kg), the aneurysm was sufficiently large for repair. The aorta was shaggy, and the superior mesenteric artery showed stenosis. In addition, the Adamkiewicz artery bifurcated 15 mm from the aneurysm and was considered at high risk of occlusion in thoracic endovascular aortic repair (TEVAR). So, we decided to treat it with open surgery. Medical history included severe chronic obstructive pulmonary disease. His forced expiratory volume in 1 s and forced vital capacity were 1.25 L/s and 2.68 L, respectively, and had a very high risk of insufficient intraoperative ventilation and postoperative respiratory complications. We were concerned that the lung would disturb the operative field to a greater extent than usual because of severe emphysema (Fig. 1), and we had to avoid the injury of the fragile lung by a rib retractor. Thus, we planned an intraoperative respiratory support system using partial bronchial blockade and venovenous extracorporeal membrane oxygenation (VV-ECMO).

**Fig. 2:**
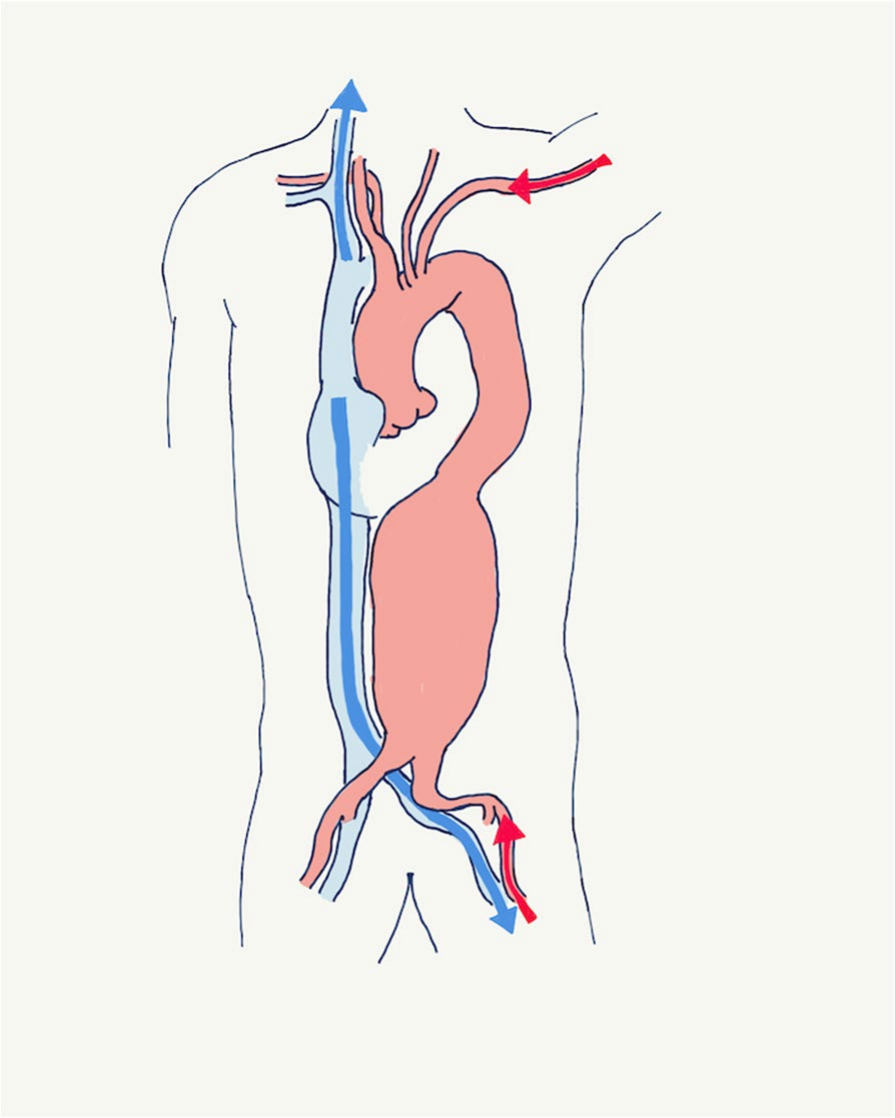
Extracorporeal circulation during an aortic repair. Red arrow: perfusion through the arterial cannulas in the left axillary and left femoral arteries. Blue arrow: drainage from the venous cannulas in the right jugular and left femoral veins

**Fig. 3:**
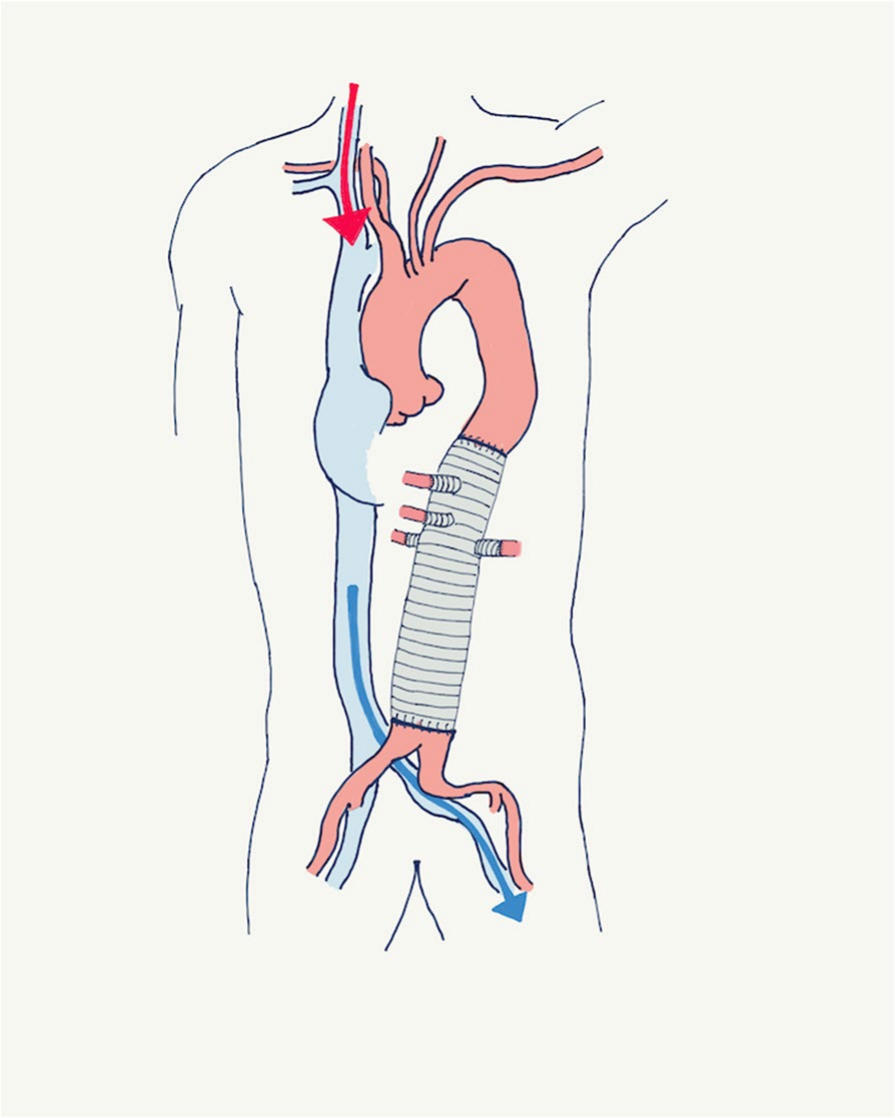
Extracorporeal circulation during hemostasis. Red arrow: perfusion through the venous cannula in the right jugular vein. Blue arrow: drainage from the venous cannula in the left femoral vein

During anesthesia induction, the bronchial blocker (BBT-B3060, DAIKEN MEDICAL Co., Osaka, Japan) was placed in the left lower lobe branch instead of the left main bronchus, and a venous cannula was placed in the superior vena cava through the right internal jugular vein. Subsequently, the patient was positioned in the right lateral decubitus position, prepped, and draped. The internal jugular venous cannula was extended with a curved tube to the operative field and connected to the venous cannula (15Fr, BioMedicus Nextgen, Medtronic, Minneapolis, MN, USA). Left axillary and femoral artery cannulas (15Fr, BioMedicus Nextgen, Medtronic, Minneapolis, MN, USA) were placed, and another venous cannula (22Fr, PCKC-V, Senko Medical Trading Co., Tokyo, Japan) was placed in the inferior vena cava through the left femoral vein (Fig. 2). Cardiopulmonary bypass (CPB) was established using the left axillary artery and two venous cannulas before making an incision. Our CPB system had an open circuit and centrifugal pump (Oxigenerator: FX-25, TERUMO, Tokyo, Japan. Pump: Revolution, LivaNova, London, UK). The pump flow rate was 3.0L/min (Perfusion Index:1.8), and we set the body temperature at 34 °C (mild hypothermia). With the left lower lobe branch blocked, one-and-a-half lung ventilation was initiated. A thoracoabdominal incision and left thoracotomy were performed. The thoracoabdominal aorta was exposed with the left thoracotomy and retroperitoneal approach. We made a limited circumference incision in the diaphragm. After the proximal and distal aortic portions were clamped, the left femoral arterial perfusion was started with the left axillary arterial perfusion. We opened the aneurysm and performed selective perfusion for all visceral branches. Following thoracoabdominal aortic replacement and reconstruction of visceral branches, the CPB was discontinued, the internal jugular venous and arterial lines were clamped and disconnected, the arterial circuit was reconnected to the internal jugular venous line, and the VV-ECMO was established using the CPB system (Fig. 3). Heparin was fully reversed with protamine, and arterial cannulas were removed. The VV-ECMO enabled the maintenance of one-and-a-half lung ventilation, which provided a clear surgical field during hemostasis. Once hemostasis was achieved, double lung ventilation was resumed, and VV-ECMO was discontinued. The operation time was 368 min, CPB time was 193 min, aortic clamping time was 121 min, selective abdominal branch perfusion time was 92 min, and VV-ECMO time was 71 min. We summarized the data related to CPB and ventilation setting (Table 1).

**Table 1:** Relationship between pump and ventilator setting and various data

**Pump**	Setting	Before CPB	CPB	VV-ECMO	After VV-ECMO
	Mean flow rate (L/m)	-	3.4	2.1	-
**Ventilator**	Setting	PCV	PCV	PCV	PCV
	PEEP (mmHg)	4	4	4	4
	Airway pressure (mmHg)	16	12	19	19
	Median tidal volume (mL)	363 (361–376)	131 (111–178)	259 (226–314)	321 (321–321)
	RR(/min)	12	8	12	12
	FiO2 (%)	100	60	100	60
**Arterial gas**	Mean PaO2 (mmHg)	561	305	425	217
	Mean PaCO2 (mmHg)	49	42	38	54

*CPB* Cardio-pulmonary bypass, *VV-ECMO* Veno-venous extracorporeal membrane oxygenation, *PCV* Pressure control ventilation, *PEEP* Positive end-expiratory pressure, *RR* Respiration rate

The patient was extubated on postoperative day one, discharged from the ICU on day three, and discharged home on day 16 without respiratory or other complications.

## Discussion and conclusions

We successfully maintained oxygenation and minimal lung inflation using CPB circuits in a patient with severe lung disease during open thoracoabdominal aortic repair with one-and-a-half lung ventilation and VV-ECMO support. In this case, double-lung ventilation would have disturbed proximal aorta exposure, and one-lung ventilation would have led to insufficient oxygenation and right lung overinflation. Excessive driving pressure is known to cause pulmonary complications, such as ventilator-induced lung injury due to barotrauma and volutrauma. Therefore, it is essential to minimize intraoperative changes in ventilation volume and avoid lung overinflation. In this particular case of type III thoracoabdominal aortic aneurysm, the left upper lobe continuous ventilation did not interfere with the surgical field, and we selectively blocked the left lower lobe.

Selective lobar blockade for cardiac surgery has been reported in several studies [4–6]. Ren et al. reported a strategy for right middle and lower lobe blockade in minimally invasive cardiac surgery. They reported that lung isolation using a bronchial blocker reduced the incidence of hypoxemia after CPB compared with conventional lung isolation, while maintaining a clear surgical field. They also mentioned the disadvantages of the lobar blockade, including difficult suction, potential dislodgement or loss of seal, and more time to acquire a suitable position for lung isolation [4]. In our case, we avoided one-and-a-half lung ventilation without mechanical support by opening the chest after establishing CPB and by using VV-ECMO after weaning from the CPB. With VV-ECMO support, both Pa02 and PaCO2 were maintained appropriately even under a restricted ventilator setting.

In patients with significant respiratory dysfunction who cannot tolerate one-lung ventilation, VV-ECMO is a useful support tool during video-assisted thoracic surgery [7, 8]. Ohsawa et al. reported two cases of VV-ECMO use in patients with chronic respiratory disease and one-lung ventilation difficulty. In one patient with a history of bilateral pneumothoraces, left lung bulla thoracoscopic resection was successfully performed with ECMO support [9]. In another case with bilateral pneumothoraces due to lymphangioleiomyomatosis, the entire lung parenchyma was thoracoscopically covered with cellulose oxide sheets and fibrin glue, while supported by VV-ECMO. In both cases, the VV-ECMO was weaned on the day of the surgery, and the ventilator was weaned on postoperative day one without any respiratory complications.

To the best of our knowledge, this is the first report on intraoperative VV-ECMO support use during open thoracoabdominal aortic aneurysm repair surgery in a patient with severe lung disease. The advantage of our strategy is the quick conversion from CPB to VV-ECMO using the same circuit. This method does not require any additional material or resources, except for the internal jugular venous cannula.

There may be concerns about potential thrombotic events caused by VV-ECMO without heparinization. Although we used an open circuit for heparin-free VV-ECMO, we did not experience any thrombotic events.

In conclusion, a combination of partial lung blockade and VV-ECMO use after weaning from CPB is an effective lung protection strategy during aortic repair, requiring thoracotomy in patients with severe lung disease.

## Data Availability

Data sharing is not applicable to this article as no datasets were generated or analyzed during the current study.

## Abbreviations

CT: Computed tomography
VV-ECMO: Venovenous extracorporeal membrane oxygenation
CPB: Cardiopulmonary bypass

## References

[1] Harky A, Othman A, Shaw M, et al. Contemporary results of open thoracic and thoracoabdominal aortic surgery in a single United Kingdom center. J Vasc Surg. 2021;73(5):1525–1532.e4. 10.1016/j.jvs.2020.09.027. 33068762 10.1016/j.jvs.2020.09.027

[2] Slinger P, Kilpatrick B. Perioperative lung protection strategies in cardiothoracic anesthesia: are they useful? Anesthesiol Clin. 2012;30(4):607–28. 10.1016/j.anclin.2012.07.001. 23089498 10.1016/j.anclin.2012.07.001

[3] Crawford ES, Coselli JS. Thoracoabdominal aneurysm surgery. Semin Thorac Cardiovasc Surg. 1991;3:300–22. 1793767

[4] Ren Y, Lyu Y, Yu Y, Jin L, Hu Y, Guo K, et al. Selective right middle and lower lobar blockade for minimally invasive cardiac surgery: a prospective, single-center, randomized controlled study. Ann Transl Med. 2021;9:254. 10.21037/atm-20-986. 33708881 10.21037/atm-20-986 PMC7940934

[5] Grocott HP, Darrow TR, Whiteheart DL, Glower DD, Smith MS. Lung isolation during port-access cardiac surgery: double-lumen endotracheal tube versus single-lumen endotracheal tube with a bronchial blocker. J Cardiothorac Vasc Anesth. 2003;17:725–7. 10.1053/j.jvca.2003.09.012. 14689413 10.1053/j.jvca.2003.09.012

[6] Agrawal DR, Nambala S, Fartado A. Selective lobar blockade in minimally invasive coronary artery bypass grafting: a technical advantage in patients with low respiratory reserve that precludes one-lung ventilation. Ann Card Anaesth. 2016;19:542–4. 10.4103/0971-9784.185560. 27397466 10.4103/0971-9784.185560 PMC4971990

[7] Gillon SA, Toufektzian L, Harrison-Phipps K, Puchakayala M, Daly K, Ioannou N, et al. Perioperative extracorporeal membrane oxygenation to facilitate lung resection after contralateral pneumonectomy. Ann Thorac Surg. 2016;101:e71–3. 10.1016/j.athoracsur.2015.08.045. 26897234 10.1016/j.athoracsur.2015.08.045

[8] Lei J, Su K, Li XF, Zhou YA, Han Y, Huang LJ, et al. ECMO-assisted carinal resection and reconstruction after left pneumonectomy. J Cardiothorac Surg. 2010;5:1–3. 10.1186/1749-8090-5-89. 20961431 10.1186/1749-8090-5-89 PMC2974678

[9] Ohsawa F, Kamiyoshihara M. Two cases of video-assisted thoracoscopic surgery combined with extracorporeal membrane oxygenation. J Jap Surgl Ass. 2020;81:243–7. 10.3919/jjsa.81.243.

